# Eggshell membrane ameliorates hepatic fibrogenesis in human C3A cells and rats through changes in PPARγ-Endothelin 1 signaling

**DOI:** 10.1038/srep07473

**Published:** 2014-12-15

**Authors:** Huijuan Jia, Wanping Aw, Kenji Saito, Manaka Hanate, Yukio Hasebe, Hisanori Kato

**Affiliations:** 1 Corporate Sponsored Research Program “Food for Life, ” Organization for Interdisciplinary Research Projects, The University of Tokyo, Tokyo, Japan; 2 Institute of Advanced Biosciences, Keio University, Yamagata, Japan; 3 Department of Applied Biological Chemistry, Graduate School of Agricultural and Life Sciences, The University of Tokyo, Tokyo, Japan; 4 ALMADO Inc., Tokyo, Japan

## Abstract

Our previous nutrigenomic findings indicate that eggshell membrane (ESM) may prevent liver fibrosis. Here we investigated the effects and mechanisms underlying ESM intervention against liver injury by using DNA microarray analysis and comparative proteomics. *In vitro* hydrolyzed ESM attenuated the TGFβ1-induced procollagen production of human hepatocyte C3A cells and inhibited the expression of Endothelin 1 (*EDN1*) and its two receptors, and extracellular matrix components. *In vivo* male Wistar rats were allocated into a normal control group, a CCl_4_ group (hypodermic injection of 50% CCl_4_ 2×/wk) and an ESM group (20 g ESM/kg diet with CCl_4_ injection) for 7 wks. Dietary ESM ameliorated the elevated activity of ALT/AST, oxidative stress and collagen accumulation in liver, accompanied by the down-regulated expression of Edn1 signaling and notable profibrogenic genes and growth factors as well as peroxisome proliferator-activated receptor gamma (PPARγ). Concomitantly, the decreased expressions of Galectin-1 and Desmin protein in the ESM group indicated the deactivation of hepatic stellate cells (HSCs). Through a multifaceted integrated omics approach, we have demonstrated that ESM can exert an antifibrotic effect by suppressing oxidative stress and promoting collagen degradation by inhibiting HSCs' transformation, potentially via a novel modulation of the PPARγ-Endothelin 1 interaction signaling pathway.

Hen eggshell membrane (ESM) is a natural material that can be easily obtained as clean, nontoxic and low-priced waste from the food industry^1^,^2^. Due to properties such as its high surface area, porous structure, inert nature, and water permeability, ESM has been used in metallurgy and bioremediation applications such as the recovery of gold from electroplating waste water^3^ and platforms for enzyme immobilization^4^. Notably, these studies did not deal with the food applications of ESM, although ESM contains high amounts of collagenized fibrous proteins.

Eggshell meal (both shell and membrane) has been officially recognized by the Association of American Feed Control Officials as a safe feed additive for both companion and livestock animals since 1982^5^. The safety of ESM as a novel dietary supplement was confirmed by Ruff et al. in 2012^5^. They also reported that ESM can suppress the production of tumor necrosis factor-alpha in cultures of peripheral blood mononuclear cells, revealing that ESM is a consumable anti-inflammatory product. As a clinical treatment, ESM was shown in human trials to maintain healthy joint and connective tissues, which may be the result of the combination of various components^6^. However, most ESM is still discarded to landfills together with eggshells, without any pretreatment^7^. In Japan, the annual generation of discarded ESM waste from food processors is estimated to be over 7,000 tons. The disposal of ESM and eggshells creates an environmental and financial burden, and alternative uses for these materials will thus be of great benefit.

In our previous work, dietary ESM treatment altered the expression of genes involved in rat liver extracellular matrix (ECM) homeostasis by down-regulating the expressions of collagen type I alpha 1 (*Col1a1*), integrin beta-like 1, decorin, asporin, and lumican^1^. Additionally, serum obtained from rats given the ESM diet suppressed the expression of *COL1A1* and *COL1A2* in human hepatocyte C3A cells^1^.

On the basis of these nutrigenomic findings obtained *in vivo* and *ex vivo*, we hypothesize that ESM in the diet may help prevent liver fibrosis, which is the main cause of liver cirrhosis. We designed the present study to investigate the beneficial effects of hydrolyzed ESM (HEM) against transforming growth factor beta 1 (TGFβ1)-induced collagen production by C3A cells. To test this hepatoprotective role *in vivo*, we subsequently used a carbon tetrachloride (CCl_4_)-induced liver injury model in rats, and the underlying mechanisms were elucidated by transcriptome profile characterization using a DNA microarray and quantitative proteomic platform using isobaric tags for relative and absolute quantification (iTRAQ) technology.

## Results

### Cytotoxicity and procollagen production in TGFβ1-treated C3A cells

**Figure 1:**
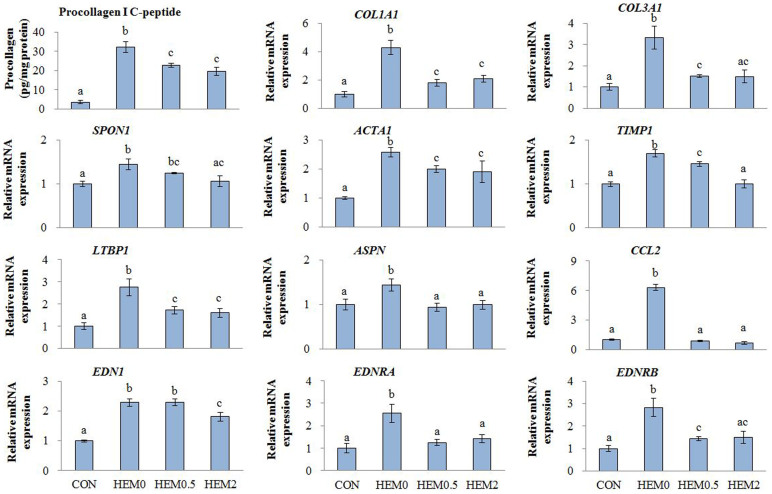
Effects of HEM on procollagen Type I C-peptide production in media, and gene expression in TGFβ1-treated C3A cells. CON, C3A cells without TGFβ1 stimulation; HEM0, without HEM addition; HEM0.5 and HEM2, 0.5 and 2 mg/mL of HEM addition, respectively. Results are means ± SE in each group (n = 3). Data with different letters (a, b, c) are significantly different at *P* < 0.05 by Dunnett's test.

After a 96-h TGFβ1 treatment, the LDH activities in the HEM0, HEM0.5 and HEM2 groups were 1.77 ± 0.03, 1.86 ± 0.12 and 1.66 ± 0.05, respectively, compared to CON at 1.00 ± 0.10. This showed no evidence of cell toxicity with either of the HEM concentrations. Procollagen Type I C-peptide production was significantly increased in the HEM0 group, and the effect was diminished by both 0.5 and 2 mg/mL of HEM supplementation (Fig. 1).

### Effects of HEM addition on cellular gene expression

The results of the real-time RT-PCR indicated that HEM supplementation significantly down-regulated the expression of the genes for actin, alpha 1 (*ACTA1*), a vital marker of hepatic stellate cell (HSC) activation, and the following ECM components and profibrogenic factors: *COL1A1*; collagen, type III, alpha 1(*COL3A1*); spondin 1, ECM protein (*SPON1*); asporin (*ASPN*); tissue metallopeptidase inhibitor 1 (*TIMP1*), as well as the potent vasoconstrictor peptide Endothelin 1 (*EDN1*) and its type A (*EDNRA*) and type B (*EDNRB*) receptors. The genes encoding latent transforming growth factor beta binding protein 1 (*LTBP1*); and chemokine (C-C motif) ligand 2 (*CCL2*) were also significantly down-regulated (Fig. 1).

### Changes in body and organ weights

**Figure 2:**
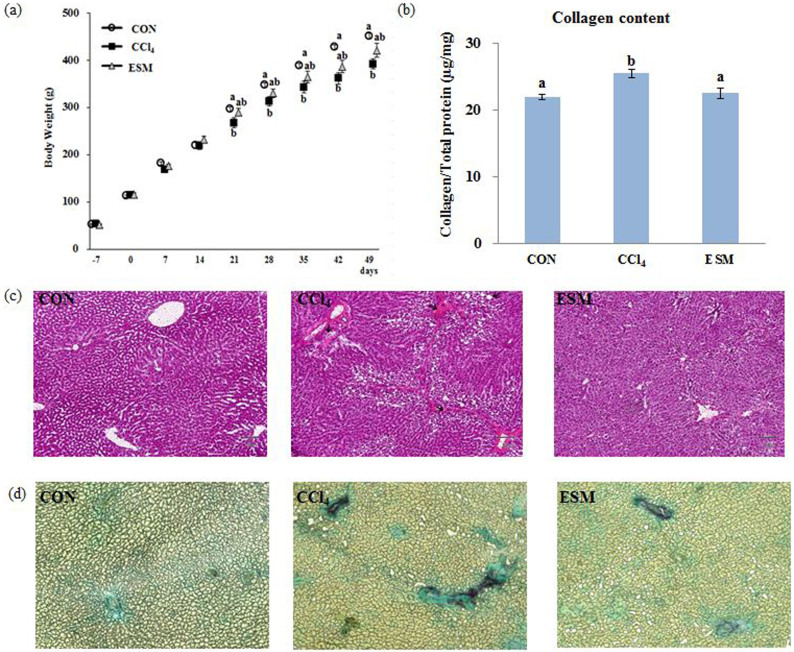
Changes in body weight (a), collagen content in liver (b), and histological features using H&E staining (c), Sirius red/Fast green staining of liver sections (d). CON, control rats; CCl_4_, rats administered only CCl_4_; ESM, rats administered CCl_4_ and ESM (20 g kg^−1^). Results are means ± SE in each group (n = 6). Data with different letters (a,b,c) are significantly different at *P* < 0.05 by Dunnett's test.

Significant body weight changes between the CON and CCl_4_ groups were observed starting from the 28th day, and this trend was maintained up to the last day with more significant differences. However, the ESM group showed a tendency for higher body weights compared to the CCl_4_ group from the 21st day, suggesting that ESM prevented the body weight decrease in the rats (Fig. 2a). There were no significant differences in food intake or organ weights between the CCl_4_ and ESM groups (Suppl. Table S2).

### Biochemical changes in liver function and lipid peroxidation contents

The liver functions measured by plasma enzyme activities are shown in Table 1. An approximate fourfold increase in AST and fivefold increase in ALT activity could be observed after 7 wks of repeated CCl_4_ injection compared to the CON rats. This marked liver damage was significantly attenuated by ESM treatment, following which the AST and ALT activities were decreased by 41% and 51%, respectively.

The CCl_4_-administered rats had elevated plasma and liver TBARS levels, indicating marked oxidative stress induced by CCl_4_. ESM treatment for 7 wks significantly normalized the plasma TBARS level compared to that of the CON group. This may be due to relatively lower lipid peroxidation in the liver. In addition, the plasma marker of liver fibrosis, ELF score, showed strong tendency (*P* = 0.07) of improved fibrotic state by dietary ESM (Table 1).

### Histopathology

Since a high TBARS level can be attributed to membrane lipid peroxidation, which is one cause of hepatic injuries as a result of CCl_4_-induced free radical production, we conducted a histological examination using H&E staining (Fig. 2c). In the CCl_4_-injected rats, the liver structure of the hepatic parenchyma and blood and bile duct was disordered. Various histological changes to the liver such as ballooning degeneration and infiltration of inflammatory cells were observed in the CCl_4_ group compared to the CON group. In contrast, the sections of the livers from rats in the ESM group show ameliorated liver structure.

### Hepatic collagen content

Fig. 2d depicts liver sections stained with Sirius red/Fast green, which is used to visualize collagen in a deep purplish red color. Our image analysis revealed that the CCl_4_ administration induced significant collagen deposition around pericentral areas which started to extend into the hepatic lobules to separate them. The ESM diet for 7 wks partially preserved the normal architecture of the parenchyma. Corroborating the histopathological analysis, the biochemical findings showed that the fibrosis index in the ESM group (22.5%, *P* < 0.05) revealed by collagen deposition was significantly lower than that in the CCl_4_ group (25.4%), and comparable to the 21.9% in the livers of the normal animals (Fig. 2b).

### Variations in the hepatic gene expression profile

**Figure 3:**
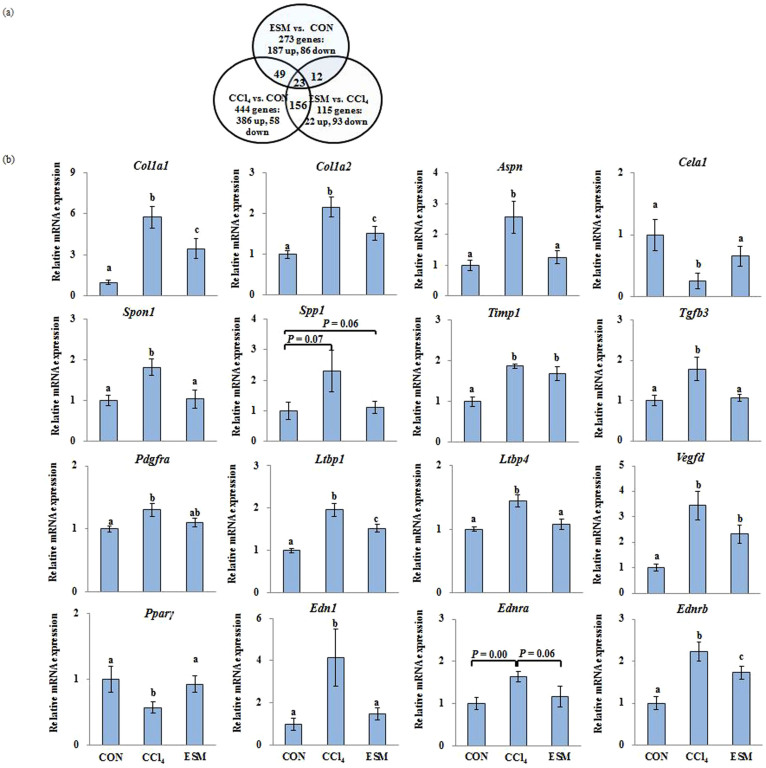
Venn diagrams of differentially expressed genes (more than 1.5-fold) in the liver (a). Each of the circles represents the genes in the respective comparison. The numbers in the spaces between overlapping circles represent the number of genes that were commonly changed in the two comparison. (b): Quantification of gene expression of key genes involved in ECM turnover and profibrogenesis. CON, control rats; CCl_4_, rats administered CCl_4_; ESM, rats given CCl_4_ and ESM (20 g kg^−1^). Results are means ± SE in each group (n = 6). Data with different letters (a,b,c) are significantly different (*P* < 0.05) by Dunnett's test.

Three pairs of comparisons were performed to identify the hepatic gene expression associated with CCl_4_ administration and the ESM diet. Among the total analyzed genes, 444 genes showed a change of more than 1.5-fold as differentially expressed genes in response to the CCl_4_ administration (Fig. 3a). In the ESM-fed rats, 22 genes were up-regulated and 93 genes were down-regulated compared to the CCl_4_ rats. As some genes had no annotation in the database, 83 genes out of the 115 probes were counted as differentially expressed genes in the rat liver. Functional categorizing and clustering for changed genes by ESM were assigned according to the IPA, which revealed ‘Hepatic Fibrosis/HSCs Activation’ as the top canonical pathway. The three highest ranked functions are involved in ‘Liver Necrosis/Cell Death,’ ‘Liver Cirrhosis,’ and ‘Liver Proliferation.’ These results indicate that the ESM treatment had a notable effect on the CCl_4_-induced injury. The list of the changed genes is further detailed in Suppl. Table S4.

Similar to the HEM's regulatory action in C3A cells, the ESM diet transcriptionally down-regulated the following multiple hepatic fibrotic gene expressions, which are involved in cytokine signaling, collagen synthesis, HSCs activation, and ECM turnover: *Col1a1*; collagen, type I, alpha 2(*Col1a2*); *Spon1*; *Aspn*; *Spp1*; *Timp1*; *Edn1*; *Ednra*; *Ednrb*; transforming growth factor beta 3 (*Tgfβ3*); *Ltbp1*; latent transforming growth factor beta binding protein 4 (*Ltbp4*), platelet-derived growth factor receptor (*Pdgfr*), and vascular endothelial growth factor D (*Vegfd*), and the ESM diet up-regulated chymotrypsin-like elastase family, member 1 (*Cela1*), a degradation protease of ECM elastin^8^, and peroxisome proliferator-activated receptor gamma (PPARγ) (Fig. 3b).

### Comparative proteome analysis by iTRAQ

**Figure 4:**
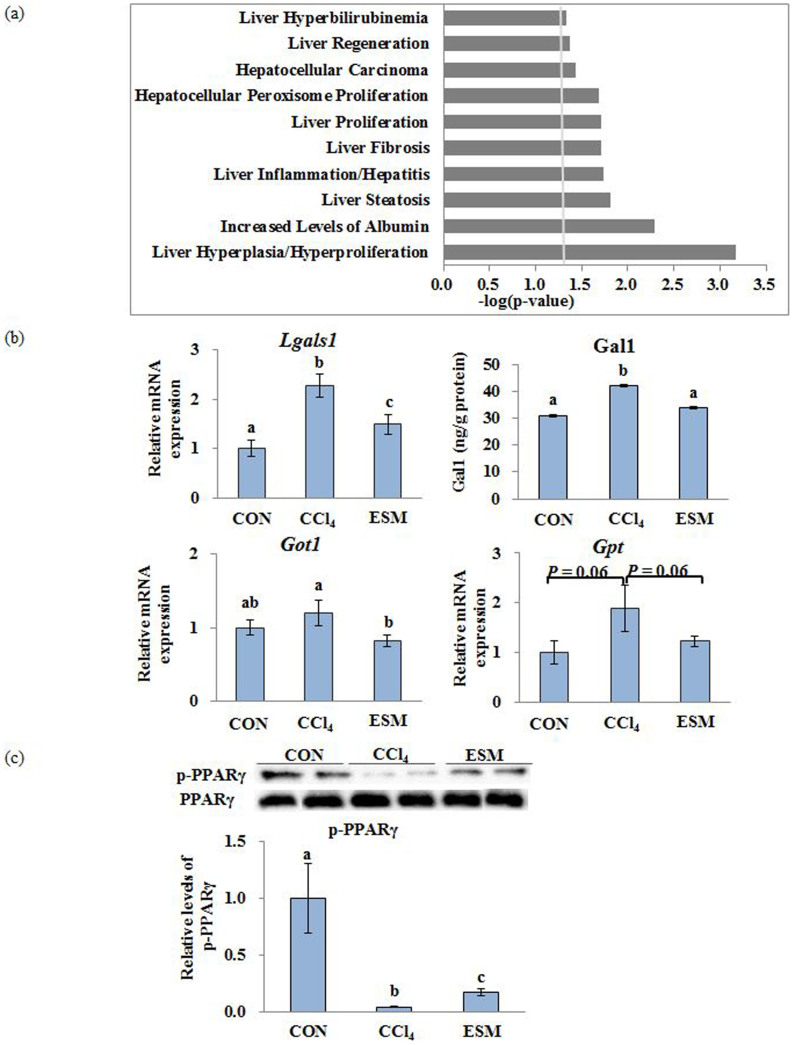
‘Liver Tox Function’ categorized by IPA (threshold > 1.25) of differentially expressed proteins by iTRAQ analysis (a). Validation of differentially expressed proteins (b). Western blotting analysis of p-PPARγ protein levels in rat liver (c). CON, control rats; CCl_4_, rats administered CCl_4_; ESM, rats given CCl_4_ and ESM (20 g kg^−1^). Results are means ± SE in each group (n = 6). Data with different letters (a,b,c) are significantly different at *P* < 0.05 by Dunnett's test.

When we classified the proteins as significantly regulated or not, we included a 1.2-fold cut-off for the 1,044 unique proteins identified at more than the 95% confidence level. According to this criterion, we screened 219 proteins, out of which 71 proteins were up-regulated and 148 proteins were down-regulated in the ESM group compared to the CCl_4_ group. These proteins were classified into 10 hepatic functional categories, in which the confidence threshold was more than 1.25 using the IPA classification system (Fig. 4a). The top five molecular function categories were ‘Liver Hyperplasia/Hyperproliferation,’ ‘Increased Levels of Albumin,’ ‘Liver Steatosis,’ ‘Liver Inflammation/Hepatitis,’ and ‘Liver Fibrosis,’ which were complementary to that of the transcriptome.

As shown in Table 2, we selected nine differential proteins which were involved mainly in ECM accumulation and HSC activity. They were all significantly reduced after the ESM diet compared to the CCl_4_ group: Galectin-1 (Gal-1, *Lgals1*), which is generated by activated HSCs and participates in galactoside binding, thereby inducing different intracellular signaling pathways leading to the proliferation of HSCs^9^,^10^; desmin, an HSC activation-related protein; Serotransferrin, which could induce a significant increase in procollagen 1(I) mRNA expression^11^; Collagen alpha-1(I) chain (Col1a1); Collagen alpha-2(I) chain (Col1a2); GTP-binding nuclear protein Ran, which is involved in many aspects of nuclear function and the *in vivo* down-regulation of Ran protein resulting in enhanced apoptosis and reduced tumor growth^12^; Solute carrier family 2, facilitated glucose transporter member 1(Glut1, *Slc2a1*), a key rate-limiting factor for the transport and metabolism of glucose, whose expression is increased in hepatocellular cancer (HCC) and which promotes the tumorigenicity of HCC cells^13^,^14^; Aspartate aminotransferase, cytoplasmic; and Casein kinase II subunit beta, a polypeptide increased in the HCC^15^. The validation of some of these differential protein expressions is shown in Fig. 4b.

### Western blotting analysis of hepatic p-PPARγ protein levels

Levels of hepatic p-PPARγ were significantly lower both in CCl_4_ and ESM groups compared to the CON group, whereas dietary ESM restored this decrease by approximately fourfold as to CCl_4_ group (Fig. 4 c).

## Discussion

Hepatic fibrosis is a dynamic process characterized by a central role of HSCs, which are activated from quiescent HSCs into a myofibroblast-like phenotype in response to hepatocyte injury, followed by the secretion and deposition of profibrogenic mediators and ECM components^16^. As such, the inhibition of HSC activation is regarded as one of the essential mechanisms to prevent fibrogenesis. In the present study, to investigate the bioavailability of ESM in the process of hepatic fibrosis, we utilized a stimulation model of TGFβ1 (which is known as a key regulator in HSC activity *in vitro*) by using soluble hydrolyzed products of ESM, and we used a CCl_4_-induced hepatic injury model *in vivo* that causes oxidative stress.

In the cellular investigation, we investigated how HEM blocked TGFβ1-induced fibrogenesis reflected by the markedly low levels of ECM components and the marker of HSC activation. With HEM supplementation, we observed significantly down-regulated expressions of a powerful vasoconstrictor peptide, EDN1, and its two receptors EDNRA and EDNRB. Under normal conditions, the sinusoidal endothelium is the major source of EDN1, which appears to be important in intrahepatic circulatory homeostasis. However, during stimulation by a number of exogenous factors such as TGFβ^17^, EDN1 synthesis shifts to not only induce stellate cell proliferation but also increases ECM production^18^ by virtue of the expression of EDNRA and EDNRB^19^. EDN1 exhibits an autocrine effect on HSCs and is involved in both HSC activation and the contractile response of HSCs to destroy the graft sinusoidal microcirculation balance^20^.

Importantly, a large body of experimental literature suggests that EDNRA and EDNRB expression correlates with the severity of the disease^21^, and therapy using an EDN receptor antagonist directed at intervening in the EDN1 signaling pathway had significant therapeutic potential in patients with liver disease^22^. Given the important role of EDN1 in fibrosis, our present findings at the cellular level suggest that HEM suppresses the EDN1 signaling network via the down-regulation of EDNRA and EDNRB expression, which in turn contributed to the cells' increased resistance to exogenous TGFβ1-stimulated fibrosis.

We subsequently comprehensively investigated the beneficial functions of ESM *in vivo*. We found that dietary ESM also exhibited potential inhibitory effects on hepatic injury and fibrosis induced by CCl_4_ in rats, as evidenced by the rats' decreased abdominal fat weights, improved plasma levels of ALT, AST, TBARS and hepatic TBARS, and the abnormal accumulation of collagen and degree of histological changes. Accordingly, we investigated the changes in hepatic gene and protein profiles related to relevant metabolic pathways to elucidate the underlying molecular mechanisms.

Consistent with the low collagen accumulation in the ESM group, significantly down-regulated ECM synthesis, degradation and turnover-related genes suggest that ESM is related to both the ECM synthesis and the degradation pathway involved in fibrosis. Although there was no significant difference in the expression of Acta1, the down-regulated HSC activation-related markers such as Gal-1 transcript and protein, desmin protein, and the mRNA levels of alpha-crystallin B, *Edn1* and its two receptors might contribute to the low proliferation and activation of HSCs and low ECM production.

HSC activation results in the secretion of several pro-fibrogenic cytokines, such as TGFβ1, platelet-derived growth factor (PDGF), vascular endothelial growth factor (VEGF) and insulin-like growth factor (IGF)^16^,^23^. TGFβ family members TGFβ1, TGFβ2 and TGFβ3 (which share approx. 70% sequence identity and exhibit distinct functions *in vivo*) are structurally homologous dimeric proteins and physiologically important in regulating multiple biological processes, including proliferation, ECM synthesis and immune response^24^,^25^.

TGFβ1 is well known to stimulate HSC proliferation and the synthesis and secretion of ECM. However, in the present study's ESM group, *Tgfβ3*, not *Tgfβ1*, was differentially down-regulated, the relevance of which is supported by our previous finding that the expression of *Tgfβ3* was 2.6-fold higher than the *Tgfβ1* expression in a CCl_4_-induced rat cirrhosis model for 19 wks^2^. It is worth noting that cells secrete TGFβs as a small latent complex (TGFβ–LAP–LTBP) composed of the mature dimeric TGFβs molecule and the two respective N-terminal pro-domains (LAP and LTBP). TGFβs remains inactive while being recruited within this complex; however, when the liver has been injured, several proteases (metalloproteases, elastase and plasmin) cleave LTBP from the complex, releasing TGFβ and LAP. In this process, LTBPs regulate the TGFβs activity by facilitating its secretion, localizing the latent TGFβs to specific sites in the ECM^26^. The down-regulation of *Tgfβ3*, *Ltbps* and the other growth factor-related genes such as *Pdgfr* and *Vegfd* may be one of the crucial mechanisms of ESM protection against HSC activation and fibrogenesis.

We further investigated the upstream regulator of the activation of HSCs in detail. We observed a link between EDN1 and PPARγ, a nuclear transcription factor that modulates the expression of various genes involved in many biological processes. PPARγ expression significantly decreased after HSC activation, in which HSCs primarily lose the vitamin A lipid droplets and become myofibroblasts^27^. The expression of PPARγ transforms HSCs back to a quiescent state, and the storage capacity of retinol palmitate fat is restored; thus the induction of PPARγ expression can significantly counteract the development of hepatic fibrosis^28^. Notably, PPARγ activators are targeted as a novel therapeutic strategy to inhibit enhanced Edn1 signaling under several stresses, for example cardiac fibrosis^29^,^30^,^31^.

Congruently with these reports, the significantly high levels of *PPARγ* mRNA expression and p-PPARγ protein, and restored expressions of *Edn1*, *Ednra* and *Ednrb* that we observed in the present ESM group suggest a modulatory role of ESM in PPARγ-Endothelin 1 signaling, HSC activation and the attenuated microcirculatory disturbance and graft function, and eventually the formation of fibrosis. This can also be supported by the down-regulation of the retinol-binding proteins (Rbps, 1.2-fold) and lecithin-retinol acyltransferases (*Lrats*, 0.8-fold). As this is a novel and interesting finding in our study, future experiments are needed to confirm this Edn1-PPARγ interaction by comparing ESM supplementation and Edn1 blockades combined with PPARγ activators to determine whether similar cellular mechanisms or their synergic action are responsible for the improved liver function.

**Figure 5:**
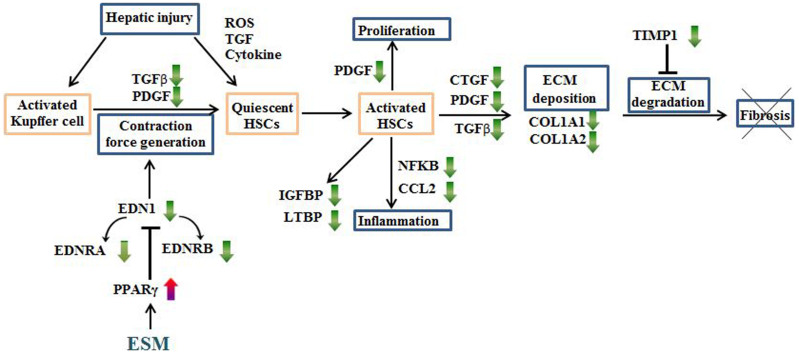
Schematic representation of the antifibrotic effects of ESM against carbon tetrachloride (CCl_4_)-induced liver injury in rats. Green and red arrows express the down- or up-regulation by dietary ESM, respectively.

Collectively, our findings indicate that ESM attenuates the enhanced HSC activation through the PPARγ-mediated repression of the Edn1 signaling pathway, thereby down-regulating growth factors, ECM components, cytokines, chemotactic factors, oxidative stress and other factors (Suppl. Table S4 and Suppl. Figure 1) simultaneously, which interact with each other forming a complex network of remodeling regulation of hepatic fibrosis. These hypothetical relationships are schematically depicted in Fig. 5.

To our knowledge, we have provided the first instance of a multi-omics study of ESM, which is expected to be impactful since the present findings not only provide information about the functions and bioavailability of ESM but also contribute to the field of environmental protection, which is increasingly important. In addition, given the concerns about impediment of Edn1 receptor antagonists in clinical trials, we propose that ESM, which is a natural, nontoxic and low-priced food waste could be a candidate for the nutritional prevention and treatment of liver fibrosis in humans. This is the most intriguing and attractive implication of this study.

In conclusion, on the basis of the nutrigenomic and proteomics information obtained by our use of a DNA microarray and a quantitative proteomic platform, ESM is a safe and natural byproduct of egg processing which possesses potent protective biochemical functions against liver injury and fibrosis. The mechanism may be associated with its tissue repair effects on the suppression of oxidative stress, improving lipid (Suppl. materials) and ECM accumulation and hepatic function, and regulating genes and proteins related to drug metabolism, the activation of HSCs and fibrogenesis by a potential novel modulation of the PPARγ-Endothelin 1 interaction signaling pathway. Through the multifaceted integrated omics approach, we have indicated that ESM may be useful for maintaining human health, especially with regard to liver fibrosis and cirrhosis.

## Methods

### Culture of C3A cells

Human C3A hepatocyte cells (CRL-12461, ATCC, Manassas, VA, USA), which are facile in producing collagen under exogenous stimulations^32^, were plated onto the inner wells of 6-well tissue culture plates at a density of 1 × 10^5^ cells per well and cultured in normal growth medium: DMEM (Sigma-Aldrich, St. Louis, MO) containing 2% fetal bovine serum and 1% penicillin/streptomycin for 24 h, then conditioned in serum-free medium with 10 mg/L transferrin (Sigma-Aldrich) and 10 mg/L insulin (Alpha Diagnostic, San Antonio, TX) for 72 h. The cells were then further conditioned in fresh serum-free medium with 500 μM sodium oleate (Sigma-Aldrich) for 48 h. Finally, the cells were exposed to 10 ng/mL TGFβ1 (Calbiochem-Merck, Nottingham, UK) and incubated for 96 h. The medium was changed every 24 h with fresh medium further supplemented with 0 (HEM0), 0.5 (HEM0.5) or 2 (HEM2) mg/mL HEM. Cells without TGFβ1 exposure were used as controls (CON). All cultures were kept at 37°C in a humidified atmosphere incubator with 5% CO_2_. Harvested cells were stored at −20°C.

Cytotoxicity under all incubation conditions was assessed by lactate dehydrogenase (LDH) leakage into the culture medium using a LDH-Cytotoxic Test kit (Wako Pure Chemical Industries, Tokyo). Procollagen Type I C-peptide was detected in 100 μL of culture medium, using an enzyme immunoassay kit (TaKaRa Bio, Shiga, Japan). Protein concentrations were determined by Bradford assay.

### Animals and experimental design

Three-wk-old male Wistar rats (50–57 g) obtained from Charles Rivers (Tokyo) were housed under controlled conditions (22 ± 1°C, 50%–60% relative humidity and 12 h light–dark cycles) with free access to tap water and diet throughout the study period. One wk after acclimatization with an American Institute of Nutrition-93G powdered diet, the rats were randomly allocated into three groups of six animals per group: the normal control group (CON), the positive control group receiving CCl_4_ (CCl_4_), and the group receiving CCl_4_ plus a 20 g kg^−1^ diet of ESM powder (ESM). Liver injury was induced in the latter two groups by a hypodermic injection of 50% CCl_4_ (in olive oil), 1 mL/kg of bodyweight 2×/wk for 7 wks. Rats in the CON group were similarly injected with olive oil. The rats' body weights were recorded 2×/wk to adjust the CCl_4_ dosage.

At the end of the 7-wk experimental period, after an overnight starvation (12 h), all of the rats were euthanized with sodium pentobarbital (1 mg/kg bodyweight i.p.). Blood samples were obtained from the carotid artery and centrifuged immediately at 800 × g for 15 min at 4°C. The collected plasma was kept at −80°C until the assays. Liver, kidney, spleen, adipose tissues and testis were excised and weighed. All of the tissues were frozen with liquid nitrogen and preserved at *−*80°C. All rats received humane care in accord with the guidelines of the Animal Usage Committee of the University of Tokyo, which gave prior approval to this study (Approval No. P13-768).

### Biochemical analysis in rats

We measured the concentrations of alanine aminotransferase (ALT), aspartate aminotransferase (AST), albumin and total protein in plasma as markers of hepatic function by using the respective assay kits (Wako, Tokyo). Lipid peroxidation parameters in the plasma and the liver, i.e., TBARS (thiobarbituric acid reactive substances) were determined spectrophotometrically with the NWLSSTM Malondialdehyde Assay kit (NWK-MDA01, Northwest Life Science Specialties, Vancouver, WA). Concentrations of plasma TIMP1, amino-terminal propeptide of type III procollagen (PIIINP) and hyaluronic acid were measured by using the ELISA (R&D Systems Inc., Minneapolis, MN, USA; Cloud-Clone Corp., Houston, TX, USA; and Biotech Trading Partners, Encinitas, CA, USA; respectively), according to manufacturer's instructions. The enhanced liver fibrosis (ELF) score was calculated using the following equation: ELF score = 2.494 + 0.846 ln (Hyaluronic acid) + 0.735 ln (PIIINP) + 0.391 ln (TIMP1)^33^.

### Liver histological assay

A portion of the left lobes of the livers was embedded in Optimal Cutting Temperature compound (Sakura Finetek, Torrance, CA), then snap-frozen in liquid nitrogen. Five-micrometer-thick sections were stained with hematoxylin and eosin (H&E) and then scanned by an Olympus BX51 microscope (Olympus Optical, Tokyo) at 100 × magnification.

### Collagen measurement in liver sections

Collagen quantification was performed by Sirius red/Fast green staining (Cosmo Bio, Tokyo; detailed in the Supplementary Methods text).

### Total RNA extraction and real-time RT-PCR

We extracted the total RNA from harvested cells and frozen rat liver by using TRIzol reagent^1^ and the RNA Isolation Kit^34^, respectively. The primers for the real-time RT-PCR analysis were designed using a web application (PRIMER3), and their sequences are given in Suppl. Table S1. The expression values for each specific gene were normalized against the GAPDH or β-actin expression levels in the cell or animal samples, respectively, and they are shown as the fold-change value of normalized mRNA amounts compared to those of the CON group.

### Hepatic cDNA microarray analysis and ingenuity® pathway analysis

We conducted a hepatic cDNA microarray analysis as described previously^1^. Affymetrix GeneChip Rat Genome 230 2.0 Arrays (Affymetrix, Santa Clara, CA) were used for the genome-wide expression profiling. We analyzed the scanned images with GeneChip Operating software (GCOS ver. 5.0, Affymetrix) to obtain the gene expression ratios between the two groups. Genes showing expression ratios of >1.5-fold were selected as differentially changed genes. Molecular interactions between genes were mapped using the Pathway Explorer function within an ingenuity pathway analysis (IPA, http://www.ingenuity.com) software.

### Proteome analysis and identification of significantly regulated proteins

The protein preparation, iTRAQ labeling, and nanoscale liquid chromatography coupled to tandem mass spectrometry (nanoLC-MS/MS) analysis were conducted as described previously^35^. We conducted the protein identification and quantification for iTRAQ samples using ProteinPilot software (ver. 4.0, AB SCIEX, Framingham, MA) with 95% confidence. The search was performed against switchProt. We used the Paragon Algorithm in the ProteinPilot software for the peptide identification and isoform specific quantification.

### Western blotting analysis

The extracted protein (25 μg) was separated electrophoretically in 4–15% polyacrylamide gradient precast gels (Mini-PROTEAN TGX; Bio-Rad Laboratories, Hercules, CA, USA) and transferred onto a PVDF membrane (GE Healthcare, Tokyo, Japan). The membrane was blocked with PVDF blocking reagent from Can Get Signal (Toyobo, Osaka, Japan), and subsequently incubated overnight at 4°C with anti-phosphorylated peroxisome proliferator-activated receptor γ (p-PPARγ) (Ser 112) (1:200 dilution, Santa Cruz Biotechnology, Santa Cruz, CA, USA) as primary antibody. After being washed with Tris-buffered saline-Tween, the membrane was incubated for 1 h with anti-rabbit IgG secondary antibody (1:5000 dilution; GE Healthcare, Tokyo, Japan). The membranes were reprobed with anti-PPARγ (H-100) (1:200 dilution) and anti-rabbit IgG (1:5000 dilution). The bands were detected with an ECL Western Blotting Detection Reagent (GE Healthcare, Tokyo, Japan) and quantified using an Ez-Capture (ATTO, Tokyo, Japan).

### Statistical analysis

The results of the cell culture and animal experiments are expressed as means ± SE of three wells or six rats, respectively. The data were analyzed with a one-way analysis of variance (ANOVA), and the Dunnett's test was used to evaluate the significant differences of the means at the level of *P* < 0.05.

## Supplementary Material

Supplementary Information Supplementary information

## Figures and Tables

**Table 1:** Biochemical changes in rat hepatic function and oxidant status

	CON	CCl_4_	ESM
AST (Karmen)	56.12 ± 2.34^a^	207.79 ± 25.0^b^	111.68 ± 17.94^c^
ALT (Karmen)	8.09 ± 0.93^a^	38.40 ± 7.81^b^	18.98 ± 4.63^c^
Albumin (g/dL)	4.70 ± 0.21	4.84 ± 0.21	4.45 ± 0.11
Total protein (g/dL)	6.95 ± 0.17	7.12 ± 0.37	6.69 ± 0.14
TBARS (μM)	3.92 ± 0.29^a^	5.11 ± 0.46^b^	3.97 ± 0.31^a^
Liver TBARS (μmol/g liver)	0.46 ± 0.02^a^	0.74 ± 0.05^b^	0.60 ± 0.03^c^
Hyaluronic acid (ng/ml)	45.28 ± 6.96^a^	77.11 ± 13.14^b^	60.30 ± 13.21^ab^
Timp1 (ng/ml)	3.74 ± 0.23^a^	5.76 ± 0.31^b^	4.81 ± 0.32^c^
PIIINP (ng/ml)	0.53 ± 0.10^a^	0.91 ± 0.11^b^	0.78 ± 0.10^ab^
ELF score	5.59 ± 0.20^a^	6.69 ± 0.24^b^	6.15 ± 0.24^ab^

CON, control rats; CCl_4_, rats administered CCl_4_; ESM, rats given CCl_4_ and ESM (20 g kg^−1^); AST, aspartate aminotransferase; ALT, alanine aminotransferase; TBARS, thiobarbituric acid reactive substances; TIMP1, tissue metallopeptidase inhibitor 1; PIIINP, amino-terminal propeptide of type III procollagen, ELF, enhanced liver fibrosis. Data are expressed as mean ± SE in each group (n = 6). Data with different letters (a,b,c) in the same column are significantly different at *P* < 0.05 by Dunnett's test.

**Table 2:** Selected potential biomarker proteins differentially expressed between CCl_4_ and ECM groups by iTRAQ analysis Partial list of proteins

Accession no.	Protein name	Gene name	CCl_4_ vs. CON (Fold change)	ESM vs. CCl_4_ (Fold change)
P12346	Serotransferrin	Tf	1.19	0.81
P11762	Galectin-1	Lgals1	1.52	0.72
P02454	Collagen alpha-1(I) chain	Col1a1	1.17	0.89
P02466	Collagen alpha-2(I) chain	Col1a2	1.14	0.94
P62828	GTP-binding nuclear protein Ran	Ran	1.58	0.81
P11167	Solute carrier family 2, facilitated glucose transporter member 1	Slc2a1	2.12	0.61
P13221	Aspartate aminotransferase, cytoplasmic	Got1	1.02	0.83
P67874	Casein kinase II subunit beta	Csnk2b	1.21	0.67
P48675	Desmin	Des	1.58	0.58

CON, control rats; CCl_4_, rats administered CCl_4_; ESM, rats given CCl_4_ and ESM (20 g kg^−1^).
