# Supplementary material for: Eggshell membrane ameliorates hepatic fibrogenesis in human C3A cells and rats through changes in PPARγ-Endothelin 1 signaling
Source: Sci Rep. 2014 Dec 15;4:7473. doi: 10.1038/srep07473 (PMC5378949; doi:10.1038/srep07473)
Supplement: Supplementary Information [file srep07473-s1.pdf]

Eggshell membrane ameliorates hepatic fibrogenesis in human C3A cells and rats through changes in PPARγ-Endothelin 1 signaling

Huijuan Jia, Wanping Aw, Kenji Saito,

Manaka Hanate, Yukio Hasebe and Hisanori Kato

Supplementary Methods

The levels of the pro-inflammatory cytokine tumor necrosis factor-alpha (TNF-α) in plasma were measured by a Quantikine enzyme-linked immunosorbent assay kit (ELISA) (R&D Systems, Minneapolis, MN, USA). The plasma levels of triglyceride (TG), total cholesterol (TC), high-density lipoprotein cholesterol (HDL), phospholipid (PL) and non-esterified fatty acid (NEFA) were determined enzymatically using commercial kits (Wako, Tokyo). Hepatic lipids were also measured using the same enzymatic kits after extraction from the frozen livers using the procedure described by Folch [1].

For the evaluation of oxidative DNA damage, 8-hydroxy-2'-deoxyguanosine (8-OHdG) levels in plasma and liver were analyzed using an ELISA kit (Japan Institute for the Control of Aging, Fukuroi, Japan) followed by total DNA extraction from liver by using a kit (Qiagen, Valencia, CA). The activities of plasma antioxidant enzymes such as superoxide dismutase (SOD), catalase (CAT) and glutathione peroxidase (Gpx) were measured using a kit (Cayman Chemicals, Ann Arbor, MI).

The principle of Sirius red/Fast green (SF) staining is the coloring of collagenous protein by Sirius red and non-collagenous proteins by fast green. The absorbance of the colors was read at 540 and 605 nm by a Microplate Reader (Multiskan JX, LabSystems, Tokyo). The collagen content of three sections for each animal was calculated using the formula below.

Fibrosis index (collagen content) = microgram collagen per milligram total protein, where:

Collagen (μg/slide) = $[\text{OD } 550 - (\text{OD } 595 \times 0.254)]/40.8 \times 1000$

Non-collagen protein (μg/slide) = $\text{OD } 605 / 2.04 \times 1000$

Total protein = Collagen + Non-collagen protein

Supplementary Results

1. General characteristics

The organ weights are listed in Supplementary Table S2. The relative liver and spleen weights were significantly increased after CCl_4 injection compared to the CON group, with no significant differences between the CCl_4 and ESM groups. The rats in the CCl_4 group had

significantly lower abdominal fat weights, and the ESM diet partially prevented this fat loss, resulting in improved body weights in the ESM group. There were no significant differences in kidney, muscle or testis weights.

No significant differences were found in the albumin and total protein levels among the three groups (Table 1). CCl_4 induced increased TNF-α concentrations that were approximately twofold higher than those of the controls, and ESM partially prevented the increase of this inflammatory cytokine, although not significantly (Suppl. Table S3).

With the CCl_4 administration, the hepatic TG, TC and PL levels were elevated and the plasma TG, TC and HDL levels were lowered (Suppl. Table 3). However, dietary ESM treatment lowered the hepatic TG and TC levels to some extent, and the plasma TG levels were slightly increased (without statistical significance) compared to those of the CCl_4 group. These results indicate that ESM partially repaired the impaired lipoprotein transport system and normalized the lipid imbalances caused by CCl_4.

In addition, the level of plasma 8-OHdG in the CCl_4-administered rats was significantly increased compared to the control rats, which may be a result of strong oxidative stress and enhanced reactive oxygen species formation. In contrast, ESM treatment attenuated the 8-OHdG levels in both tissues, although not significantly. Unexpectedly, the ESM treatment had no effect on SOD, CAT or Gpx activity (Suppl. Table 3).

2. Variations in lipid metabolism and the expressions of stress-related genes

Fatty acid synthase (Fas), fatty acid binding protein 4 (Fabp4), lipoprotein lipase (Lpl) and fatty acid translocase CD36 (Fat/CD36), which are associated with fatty acid metabolism, were down-regulated with ESM treatment. In contrast, cytochrome P450 7a1 (Cyp7a1) expression was significantly up-regulated in the rats fed ESM. In addition, one of the upstream genes of cyp7a1, farnesoid X receptor (Fxr) also showed up-regulation (1.2-fold).

In addition**,** Heat shock 70kD protein 1A1 (*Hspa1a1*), which is induced in response to various stresses; cytochrome P450, family 2, subfamily b, polypeptide 1 (*Cyp2b1*), which is related to drug metabolism; and some inflammatory genes such as S100 calcium binding protein A11 (*S100a11)* and *Ccl2* were down-expressed in the ESM group. These changes were validated by a quantitative real-time RT-PCR and were generally consistent with the two techniques (Suppl. Figure).

Supplementary Discussion

One of the earliest manifestations of CCl_4-induced liver injury is the accumulation of lipids, as shown in the plasma and hepatic lipid profiles. This results from an imbalance among the hepatic fatty acid flow, triglycerol synthesis, and excretion. The results of the genomics approach showed that three genes associated with fatty acid metabolism (*Fas*, an enzyme that catalyzes fatty acid synthesis, *Fabp4,* which plays an important role in the

storage and transport of fatty acids into the liver, and *Lpl*, a rate-limiting enzyme for the intravascular hydrolysis of lipoprotein-rich triglyceride particles) were down-regulated with ESM treatment. Decreased fatty acid synthesis and uptake as well as lipoprotein hydrolysis may thus have contributed to the low hepatic fat accumulation in the ESM group.

The up-regulated expression of *Cyp7a1*, a rate-limiting enzyme in the conversion of cholesterol to bile acids [2], indicates the influence of ESM on lipid cholesterol metabolic outputs in the liver. Additionally, one of the upstream genes of *Cyp7a1*, farnesoid X receptor (*Fxr*) also showed up-regulation. *Fxr* activation will not only help release the bile acids overload in liver, but it can prevent bile acid-induced cell death and other deleterious effects on normal liver repair pathways [3]. In this respect, by promoting regeneration and preventing cell death, ESM up-regulated *Fxr* and *Cyp7a1* are essential to promote liver repair after CCl_4-induced injury. However, the up-regulation of *Cyp7a1* was confirmed by PCR with high individual variability, and thus no significant difference was observed in the hepatic TC levels of the ESM group.

As oxidative stress plays a critical role in the activation of HSCs during liver fibrosis, and because our results showed that ESM reduced the elevated contents of TBARS and 8-OHdG, we checked the expression of genes involved in oxidative stress. Glutathione peroxidase, cytochrome b-245, alpha polypeptide, glutamate-cysteine ligase and more, were all up-regulated with CCl_4 but not changed by ESM, except for *Cyp2b1* and *Hspa1a1*, which were down-regulated following the ESM diet. Heat shock proteins are induced in response to various stresses and correlate with the degree of damage [4]. CYP2B1, one of the cytochrome P450 enzymes, can activate CCl_4 to form the free radical ($CCl_3^{\cdot}$), ($CCl_3OO^{\cdot}$) which in turn results in lipid peroxidation, DNA damage and protein denaturation [5]. After propagation of the peroxidation process, lipids are finally degraded in small molecules such as TBARS. Similarly, damaged DNA, e.g., 8-OHdG will be produced. With the ESM diet, the down-regulation of *Cyp2b1* and *Hspa1a1* indicated that less oxidative stress $CCl_3^{\cdot}$ radical formation, and resulted in lower TBARS levels. However, this decreased lipid peroxidation in the ESM group was not associated with anti-oxidative enzyme activity, as the SOD, CAT, Gpx activities and their expressions were not affected by ESM. Additionally, the ESM group showed signs of attenuated liver inflammation as indicated by decreased plasma serum AST and ALT activities. The protein and transcriptional down-regulation in ALT/AST levels may contribute to the decreased ALT/AST, which indicated that the chronic injury induced by CCl_4 was repaired.

In addition, the autocrine signaling by PDGF, which binds to and activates PDGF receptor (Pdgfr), is also regarded as one of the potent mitogens and chemotactics for HSCs, as PDGF can induce the proliferation of ECM-producing cells mediated by stimulating ERK activity [6, 7]. Moreover, IGF binding proteins (IGFBPs) can activate HSCs and increase the ECM, which is associated with the TGFβ1/Smad3 signaling pathways [8, 9]. The

overexpression of VEGFD, an important fibrogenic and angiogenic factor involved in fibrosis, has a stimulatory effect on collagen production by activated HSCs. This can occur independently of TGFβ1 overexpression [10], which may give an alternative explanation for the lack of a change in *Tgfβ1* expression. As such, the antifibrogenic activity of ESM may therefore also be attributed to its inhibitory effect on Igfbp, Pdgfr and Vegfd expression, and the inactivation of HSCs.

PPARγ has several inhibitory effects on inflammation, including the reduction of the transcriptional activities of NF-kB, a redox-sensitive transcription factor that transactivates the promoters of many types of inflammation, infection and stress genes, including cytokines. It is also known that the key to HSC proliferation (which is stimulated by oxidative stress) is the activation of NF-kB, gene expression of which is induced only in activated, but not in quiescent HSCs [11]. In the present study, the expressions of NF-kB and a monocyte-attracting chemokine, Ccl_2 (which is involved in inflammation and regulated by NF-kB) were significantly decreased in the ESM group. Therefore, the antifibrogenetic effect of ESM is due, at least in part, to an up-regulation of PPARγ and decreased NF-kB activities on inactivating matrix-producing HSCs, thereby blocking or attenuating oxidative stress, inflammatory processes and fibrosis progression. Further studies are necessary to elucidate the underlying molecular mechanisms involved in this process.

References

1. Folch J, Lees M, Sloane-Stanley GH. A simple method for the isolation and purification of total lipides from animal tissues. *J Biol Chem* **226**, 497-509 (1957).

2. Guo J*, et al.* A new TCM formula FTZ lowers serum cholesterol by regulating HMG-CoA reductase and CYP7A1 in hyperlipidemic rats. *Journal of Ethnopharmacology* **135**, 299-307 (2011).

3. Meng Z*, et al.* FXR Regulates Liver Repair after CCl4-Induced Toxic Injury. *Molecular Endocrinology* **24**, 886-897 (2010).

4. Domitrović R, Jakovac H, Milin Č, Radošević-Stašić B. Dose- and time-dependent effects of luteolin on carbon tetrachloride-induced hepatotoxicity in mice. *Experimental and Toxicologic Pathology* **61**, 581-589 (2009).

5. Knockaert L*, et al.* Carbon tetrachloride-mediated lipid peroxidation induces early mitochondrial alterations in mouse liver. *Laboratory Investigation* **92**, 396-410 (2011).

6. Qiang H*, et al.* Differential expression genes analyzed by cDNA array in the regulation of rat hepatic fibrogenesis. *Liver Int* **26**, 1126-1137 (2006).

7. Yasuda Y*, et al.* (−)-Epigallocatechin gallate prevents carbon tetrachloride-induced rat hepatic fibrosis by inhibiting the expression of the PDGFRβ and IGF-1R. *Chemico-Biological Interactions* **182**, 159-164 (2009).

8. Liu LX, Zhang HY, Zhang QQ, Guo XH. Effects of insulin-like growth factor binding protein-related protein 1 in mice with hepatic fibrosis induced by thioacetamide. *Chin Med J (Engl)* **123**, 2521-2526 (2010).

9. Liu L-X. Insulin-like growth factor binding protein-7 induces activation and transdifferentiation of hepatic stellate cellsin vitro. *World Journal of Gastroenterology* **15**, 3246 (2009).

10. Shi H, Dong L, Bai Y, Zhao J, Zhang Y, Zhang L. Chlorogenic acid against carbon tetrachloride-induced liver fibrosis in rats. *European Journal of Pharmacology* **623**, 119-124 (2009).

11. Chávez E*, et al.* Resveratrol prevents fibrosis, NF-κB activation and TGF-β increases

induced by chronic CCl4 treatment in rats. *Journal of Applied Toxicology* **28**, 35-43 (2008).

Supplementary Table S1. The primer sequences for RT-PCR

A. Human primers

Gene	Accession no	Primer sequence		Probe Set ID
ACTA1	NM_001100	Forward	CGACATCAGGAAGGACCTGT	203872_at
		Reverse	CCGATCCACACCGAGTATTT	
ASPN	NM_017680	Forward	AAGAGCTGGTTTGGGGCTAT	219087_at
		Reverse	CCAAGCAAGGTCTTCCAAAG	
CCL2	S69738	Forward	GCAATTTCCCCAAGTCTCTG	216598_s_at
		Reverse	CCCTAGCTTTCCCCAGACAT	
COL1A1	BE221212	Forward	AACAGCCGCTTCACCTACAG	1556499_s_at
		Reverse	TGGGATGGAGGGAGTTTACA	
COL3A1	AI813758	Forward	CACCTTCATTTGACCCCATC	201852_x_at
		Reverse	TACGGCAATCCTGAACTTCC	
EDN1	J05008	Forward	CCGGCTAATGAAAGAGGTTG	222802_at
		Reverse	TTGACAGGCAAAACAAAGCA	
EDNRA	NM_001957	Forward	CGCCAGACAGATTGCTGATA	204464_s_at
		Reverse	TATGATGCGCCAGTGGAATA	
EDNRB	M74921	Forward	GTCATGCTTATGCTGCTGGT	204271_s_at
		Reverse	CGGAAGTTGTCATATCCGTGA	
GAPDH	AK026525	Forward	CTCATGACCACAGTCCATGC	217398_x_at
		Reverse	CTCATGACCACAGTCCATGC	
LTBP1	NM_000627	Forward	TGCAGTTCCTTGGCTACTGTT	202729_s_at
		Reverse	CTGCTCCACAGGACAGACAA	
SPON1	AB018305	Forward	GACAGCAGATTCCCCACATT	209437_s_at
		Reverse	ACCCAACATCAATGCTCCTC	

TIMP1	NM_003254	Forward	CTGTTGTTGCTGTGGCTGAT	201666_at
		Reverse	CATCCCCTAAGGCTTGGAAC	

B. Rat primers

Gene	Accession no	Primer sequence		Probe Set ID
Actb	NM_134326	Forward	TTGCTGACAGGATGCAGAAG	1367555_at
		Reverse	GTACTTGCGCTCAGGAGGAG	
Aspn	NM_001014008	Forward	AGGAAAGCCCTTTGGAAGAG	1380726_at
		Reverse	TTCCAGGGATTACCTAATGTGC	
Ccl2	NM_031530	Forward	CAAGAGAATCACCAGCAGCA	1367973_at
		Reverse	CCTTATTGGGGTCAGCACAG	
Cela1	NM_012552	Forward	CTTGGTGAACGGCCAGTATT	1387819_at
		Reverse	GCCACTGGACTCAGGAAGAC	
Col1a1	NM_053304	Forward	GGGCAAGACAGTCATCGAAT	1388116_at
		Reverse	AGATTGGGATGGAGGGAGTT	
Col1a2	NM_053356	Forward	CTCCAAGGAAATGGCAACTC	1370155_at
		Reverse	CAATGCTGTTCTTGCAGTGG	
Cyp2b1	NM_001134844	Forward	CCCAAGGACATTGACCTCAC	1371076_at
		Reverse	TTCAGTGCCATTCACAGGAA	
Cyp7a1	NM_012942	Forward	ATTCTTGTGCGGTGATGGTT	1368458_at
		Reverse	ATGTGCCTTCCCTCCAAGTA	
Edn1	NM_012548	Forward	ACCTGTCTTCGTTTGCATCC	1369519_at
		Reverse	GCCTGAGTCAGACACGAACA	
Ednra	1383641_at	Forward	TGCCACTGAGTAACACACACC	BF414702
		Reverse	GTTCCTTGTCGGAAATGTGG	

Ednrb	X57764	Forward	CGCTCTGTATTTGGTGAGCA	1387146_a_at
		Reverse	GGAGCGGAAGTTGTCGTATC	
Fabp4	NM_053365	Forward	AAATCACCCCAGATGACAGG	1368271_a_at
		Reverse	TCGACTTTCCATCCCACTTC	
Fasn	NM_012598	Forward	TCAAATTGCTGCTTGGGTTT	1367708_a_at
		Reverse	GGGACAGCATCAAGAGCATT	
Got1	NM_012571	Forward	CCGGATTCTGACCATGAGAT	1368272_at
		Reverse	GATGTGCTTCTCGTTGACCA	
Gpt	NM_031039	Forward	TGTGCCTCCTGGAAGAGACT	1387052_at
		Reverse	TGTTGCGTCAGAGACTGTCC	
Hspa1a	NM_031971	Forward	TCGAGGAGGTGGATTAGAGG	1368247_at
		Reverse	TAAGAATCGTGCACCAGCAG	
Lgals1	NM_019904	Forward	GTTGAACCTGGGGAAAGACA	1367628_at
		Reverse	AGCTTGATGGTCAGGTCAGC	
Lpl	NM_012598	Forward	TCACCAGCATCCCCATTATT	1386965_at
		Reverse	CACAACAGCGTTTCCAGTGT	
Ltbp1	NM_021587	Forward	CTTGGTCCGGAGACTTTGAA	1367912_at
		Reverse	ATCCAATTGACAGGCAATCC	
Ltbp4	NM_001170336	Forward	TGTGATTGTTTCGACGGCTA	1371500_at
		Reverse	CGGAAGGAACCATCAGTGTT	
Pdgfra	NM_012802	Forward	CCAACATGGTGGTGTGGTAA	1370941_at
		Reverse	CATGAACACGGGTATCTGGA	
Pparγ	NM_013124	Forward	CGAGGACATCCAAGACAACC	1369179_a_at
		Reverse	TCAGCGACTGGGACTTTTCT	
S100a11	NM_001004095	Forward	GATGCATCGAGTCCCTGATT	1375170_at

		Reverse	TAGCTGCCCATCACTGTTGA	
Spon1	NM_172067	Forward	AGCACCCTAAACCCAGGAGT	1370312_at
		Reverse	AAAGGATGTGGTGGTGCTTT	
Spp1	NM_012881	Forward	GATCGATAGTGCCGAGAAGC	1367581_a_at
		Reverse	CTTGTCCTCATGGCTGTGAA	
Tgfb3	NM_013174	Forward	CTAGACACCTTCCGGGTCAG	1367859_at
		Reverse	GTCTAGGGCAGGAGGGAAAC	
Timp1	NM_053819	Forward	CATGGAGAGCCTCTGTGGAT	1367712_at
		Reverse	TCAGATTATGCCAGGGAACC	
Vegfd	NM_031761	Forward	GGTGATTCCCCAATTCACTG	1373882_at
		Reverse	CAGGCAACCTTTTCTCGTTC	

Supplementary Table S2. Changes in food intake and tissue weights (%)

	CON	CCl_4	ESM
Total food intake (kg)	1.00 ± 0.04[a]	0.89 ± 0.03[b]	0.91 ± 0.11[b]
Liver	2.65 ± 0.03[a]	3.16 ± 0.13[b]	2.91 ± 0.07[b]
Kidney	0.63 ± 0.04	0.61 ± 0.02	0.58 ± 0.02
Spleen	0.22 ± 0.02[a]	0.28 ± 0.01[b]	0.29 ± 0.01[b]
Gastrocnemius muscle	0.58 ± 0.01	0.63 ± 0.02	0.61 ± 0.01
Testis	0.95 ± 0.07	0.97 ± 0.03	0.89 ± 0.04
Epididymal fat	2.27 ± 0.21[a]	1.65 ± 0.09[b]	2.05 ± 0.22[a,b]
Perirenal fat	1.87 ± 0.23	1.58 ± 0.19	1.96 ± 0.15
Omental fat	1.40 ± 0.13	1.27 ± 0.11	1.47 ± 0.08
Abdominal fat	5.54 ± 0.50[a]	4.50 ± 0.31[b]	5.48 ± 0.27[a]

CON, control rats; CCl_4, rats administered CCl_4; ESM, rats administered CCl_4 and ESM (20 g kg^{-1}). Data are mean ± SE in each group (n=6). Data with different letters (a,b,c) in the same column are significantly different at $P < 0.05$ by Dunnett's test.

Supplementary Table S3. Biochemical changes

	CON	CCl_4	ESM
TNF-α level (pg/mL)	45.20 ± 4.82 [a]	97.13 ±11.13 [b]	82.88 ± 4.13 [b]
8-OHdg (ng/mL)	0.055 ± 0.013[a]	0.095 ± 0.008[b]	0.085 ± 0.013[ab]
SOD activity (U/dL)	69.08 ± 6.61	54.75 ± 6.73	53.46 ± 4.52
CAT activity (µM)	41.56 ± 4.26 [a]	26.19 ± 3.02[b]	29.25 ± 3.34[b]
Gpx activity (nmol/min/mL)	3.08 ± 0.42	2.27 ± 0.29	2.42 ± 0.07
Liver 8-OHdg (µg/g liver DNA)	1.43 ± 0.28	3.05 ± 0.67	2.34 ± 0.45
Plasma lipid			
TG (g/dL)	82.72 ± 13.26	68.37 ± 7.83	86.81 ± 13.26
TC (g/dL)	73.01 ± 7.43	67.13 ± 2.75	65.11 ± 2.75
PL (g/dL)	110.61 ± 7.92	119.68 ± 8.84	125.66 ± 6.19
HDL (g/dL)	56.59 ± 6.47[a]	35.39 ± 4.12[b]	39.40 ± 1.75[b]
NEFA (Eq/L)	0.65 ± 0.07	0.73 ± 0.08	0.63 ± 0.03
Hepatic lipid			
Hepatic TG (mg/g)	20.16 ± 1.53[a]	62.98 ± 3.78[b]	57.91 ±5.69[b]
Hepatic TC (mg/g)	3.71 ± 0.21[a]	6.71 ± 0.20[b]	6.54 ± 0.34[b]
Hepatic PL (mg/g)	8.69 ± 1.91[a]	13.47 ± 0.86[b]	13.91 ± 0.50[b]
Hepatic HDL (mg/g)	4.14 ± 0.37	4.79 ± 0.29	4.83 ± 0.39
Hepatic NEFA (mg/g)	8.07 ± 0.93	9.03 ± 0.83	8.15 ± 1.00

CON, control rats; CCl_4, rats administered CCl_4; ESM, rats administered CCl_4 and ESM (20 g kg^{-1}); 8-OHdG, 8-hydroxy-2'-deoxyguanosine; SOD, superoxide dismutase; CAT, catalase; Gpx, glutathione peroxidase; HDL, high-density lipoprotein cholesterol; TG, triglyceride; TC, total cholesterol; PL, phospholipid and NEFA, non-esterified fatty acid.

Data are mean ± SE in each group (n=6). Data with different letters (a,b,c) in the same column are significantly different at $P < 0.05$ by Dunnett's test.

Supplementary Table S4. Differentially expressed genes in rat liver

Up-regulated genes

Probe Set	Symbol	Gene Title	Fold change	
			CCl_4 vs. CON	ESM vs. CCl4
1370711_a_at	*Nupl1*	nucleoporin like 1	0.41	2.83
1374367_at	*Grxcr1*	glutaredoxin, cysteine rich 1	0.76	2.83
1387819_at	*Cela1*	chymotrypsin-like elastase family, member 1	0.31	2.14
1392189_at	*Rfx4*	Regulatory factor X, 4 (influences HLA class II expression)	0.41	2.14
1370491_a_at	*Hdc*	histidine decarboxylase	0.62	2.00
1368458_at	*Cyp7a1*	cytochrome P450, family 7, subfamily a, polypeptide 1	1.62	1.87
1370394_at	*IgG-2a*	gamma-2a immunoglobulin heavy chain	1.07	1.87
1370778_at	*Mup5*	major urinary protein 5	1.07	1.74
1384007_at	*Atl1*	atlastin GTPase 1	0.76	1.74
1368155_at	*Cyp2c12*	cytochrome P450, family 2, subfamily c, polypeptide 12	0.87	1.62
1370150_a_at	*Thrsp*	thyroid hormone responsive	0.87	1.62
1375043_at	*Fos*	FBJ osteosarcoma oncogene	0.87	1.62
1377209_at	*Klhl25*	kelch-like 25 (Drosophila)	0.81	1.62
1372665_at	*Psat1*	phosphoserine aminotransferase 1	0.76	1.52
1382363_at	*Mpp5*	membrane protein, palmitoylated 5	0.93	1.52
1390284_at	*Ccdc77*	coiled-coil domain containing 77	0.87	1.52
1369179_a_at	*Pparg*	peroxisome proliferator-activated receptor gamma	0.7	1.23

Down-regulated genes

Probe Set	*Symbol*	Gene Title	CCl_4 vs. CON	ESM vs. CCl_4
1371942_at	*Gstt3*	glutathione S-transferase, theta 3	3.48	0.35
1369864_a_at	*Sds*	serine dehydratase	1.32	0.44

1370583_s_at	*Abcb1a /1b*	ATP-binding cassette, sub-family B (MDR/TAP), member 1A / 1B	3.03	0.44
1370912_at	*Hspa1a*	heat shock 70kD protein 1A	4.00	0.44
1388694_at	*RT1-T24-3*	RT1 class I, locus T24, gene 3	2.00	0.44
1390672_at	*Rprm*	reprimo, TP53 dependent G2 arrest mediator candidate	0.50	0.44
1391262_at	*Senp5*	Sumo1/sentrin/SMT3 specific peptidase 5	0.71	0.44
1368247_at	*Hspa1a /1b*	heat shock 70kD protein 1A / 1B	4.00	0.47
1368007_at	*Dmbt1*	deleted in malignant brain tumors 1	1.52	0.50
1371111_at	*RT1-EC2*	RT1 class Ib, locus EC2	1.15	0.50
1373882_at	*Figf*	c-fos induced growth factor	2.64	0.50
1376100_at	*Tubb6*	tubulin, beta 6	2.83	0.50
1383291_at	*C7 /// Tubb2c*	complement component 7 /// tubulin, beta 2c	4.92	0.50
1386965_at	*Lpl*	lipoprotein lipase	4.29	0.50
1387011_at	*Lcn2*	lipocalin 2	2.83	0.50
1370312_at	*Spon1*	spondin 1, extracellular matrix protein	2.00	0.54
1370956_at	*Dcn*	decorin	3.25	0.54
1390781_at	*Abcb10*	ATP-binding cassette, sub-family B (MDR/TAP), member 10	1.52	0.54
1367859_at	*Tgfb3*	transforming growth factor, beta 3	2.0	0.54
1370026_at	*Cryab*	crystallin, alpha B	2.46	0.54
1367568_a_at	*Mgp*	matrix Gla protein	5.66	0.57
1370445_at	*Pla1a*	phospholipase A1 member A	3.03	0.57
1372219_at	*Tpm2*	tropomyosin 2, beta	1.41	0.57
1377353_a_at	*Tnfsf13*	tumor necrosis factor (ligand) superfamily, member 13	1.74	0.57
1377907_at	*Snrnp48*	small nuclear ribonucleoprotein 48k (U11/U12)	1.00	0.57
1380726_at	*Aspn*	Asporin	2.30	0.57

1388557_at	*Tubb2c*	tubulin, beta 2c	4.29	0.57
1398373_at	*B3galnt1*	beta-1,3-N-acetylgalactosaminyltransferase 1	6.96	0.57
1368671_at	*Srpx*	sushi-repeat-containing protein, X-linked	2.00	0.62
1370155_at	*Col1a2*	collagen, type I, alpha 2	2.30	0.62
1371500_at	*Ltbp4*	latent transforming growth factor beta binding protein 4	1.62	0.62
1371527_at	*Emp1*	epithelial membrane protein 1	2.30	0.62
1371700_at	*Mfap4*	microfibrillar-associated protein 4	5.66	0.62
1372615_at	*Aoc3*	amine oxidase, copper containing 3 (vascular adhesion protein 1)	1.52	0.62
1373223_at	*Fam171b*	family with sequence similarity 171, member B	2.00	0.62
1374204_at	*Wsb1*	WD repeat and SOCS box-containing 1	1.00	0.62
1374235_at	*Rcan2*	regulator of calcineurin 2	1.15	0.62
1387609_at	*Car5a*	carbonic anhydrase 5a, mitochondrial	1.15	0.62
1392965_a_at	*Smoc2*	SPARC related modular calcium binding 2	4.29	0.62
1367581_a_at	*Spp1*	secreted phosphoprotein 1	1.41	0.66
1367712_at	*Timp1*	TIMP metallopeptidase inhibitor 1	1.74	0.66
1367894_at	*Insig1*	insulin induced gene 1	2.14	0.66
1367912_at	*Ltbp1*	latent transforming growth factor beta binding protein 1	1.87	0.66
1368160_at	*Igfbp1*	insulin-like growth factor binding protein 1	2.83	0.66
1368187_at	*Gpnmb*	glycoprotein (transmembrane) nmb	2.83	0.66
1368271_a_at	*Fabp4*	fatty acid binding protein 4, adipocyte	3.03	0.66
1368778_at	*Slc6a6*	solute carrier family 6 (neurotransmitter transporter, taurine), member 6	0.93	0.66
1370056_at	*Ly6c*	Ly6-C antigen	1.87	0.66
1370156_at	*Prnp*	prion protein	1.23	0.66
1371537_at	*B4galt5*	UDP-Gal:betaGlcNAc beta 1,4-galactosyltransferase, polypeptide	1.41	0.66

		5		
1372658_at	*Synm*	synemin, intermediate filament protein	1.00	0.66
1373135_at	*Aarsd1*	alanyl-tRNA synthetase domain containing 1	1.07	0.66
1373718_at	*Tubb2a*	tubulin, beta 2a	1.41	0.66
1375170_at	*S100a11*	S100 calcium binding protein A11 (calizzarin)	1.74	0.66
1379677_at	*Tnfsf13*	tumor necrosis factor (ligand) superfamily, member 13	1.52	0.66
1382021_at	*Pkd2*	polycystic kidney disease 2 homolog (human)	1.62	0.66
1382984_at	*Tor1b*	torsin family 1, member B	1.52	0.66
1384274_at	*LOC367746*	similar to Spindlin-like protein 2 (SPIN-2)	0.38	0.66
1385211_at	*LOC100361585*	rCG31991-like	0.93	0.66
1385247_at	*Ugt2b*	UDP glycosyltransferase 2 family, polypeptide B	0.87	0.66
1385248_a_at	*Ogn*	osteoglycin	1.52	0.66
1387854_at	*Col1a2*	collagen, type I, alpha 2	2.64	0.66
1388199_at	*Epcam*	epithelial cell adhesion molecule	1.15	0.66
1388792_at	*Gadd45g*	growth arrest and DNA-damage-inducible, gamma	0.81	0.66
1391635_at	*Ctdspl*	CTD (carboxy-terminal domain, RNA polymerase II, polypeptide A) small phosphatase-like	1.07	0.66
1391701_at	*Myst3*	MYST histone acetyltransferase (monocytic leukemia) 3	1.15	0.66
1393060_at	*Adamtsl2*	ADAMTS-like 2	2.83	0.66
1398484_at	*RGD1308221*	similar to TBC1 domain family, member 8; vascular Rab-GAP/TBC-containing	1.62	0.66
1367973_at	*Ccl2*	chemokine (C-C motif) ligand 2	2.6	0.66
1387146_a_at	*Ednrb*	endothelin receptor type B	2.5	0.66
1369519_at	*Edn1*	endothelin 1	1.6	0.71
1370941_at	*Pdgfra*	platelet derived growth factor receptor, alpha polypeptide	1.6	0.76

1388116_at	*Col1a1*	collagen, type I, alpha 1	1.6	0.76
1367708_a_at	*Fasn*	fatty acid synthase	1.1	0.76
1387052_at	*Gpt*	glutamic-pyruvate transaminase (alanine aminotransferase)	1.6	0.76
1368272_at	*Got1*	glutamic-oxaloacetic transaminase 1, soluble (aspartate aminotransferase 1)	1.2	0.81
1371076_at	*Cyp2b1* /// *Cyp2b2*	cytochrome P450, family 2, subfamily b, polypeptide 1 /// cytochrome P450, family 2, subfamily b, polypeptide 2	1.2	0.87
1383641_at	*Ednra*	endothelin receptor type A	1.1	0.87

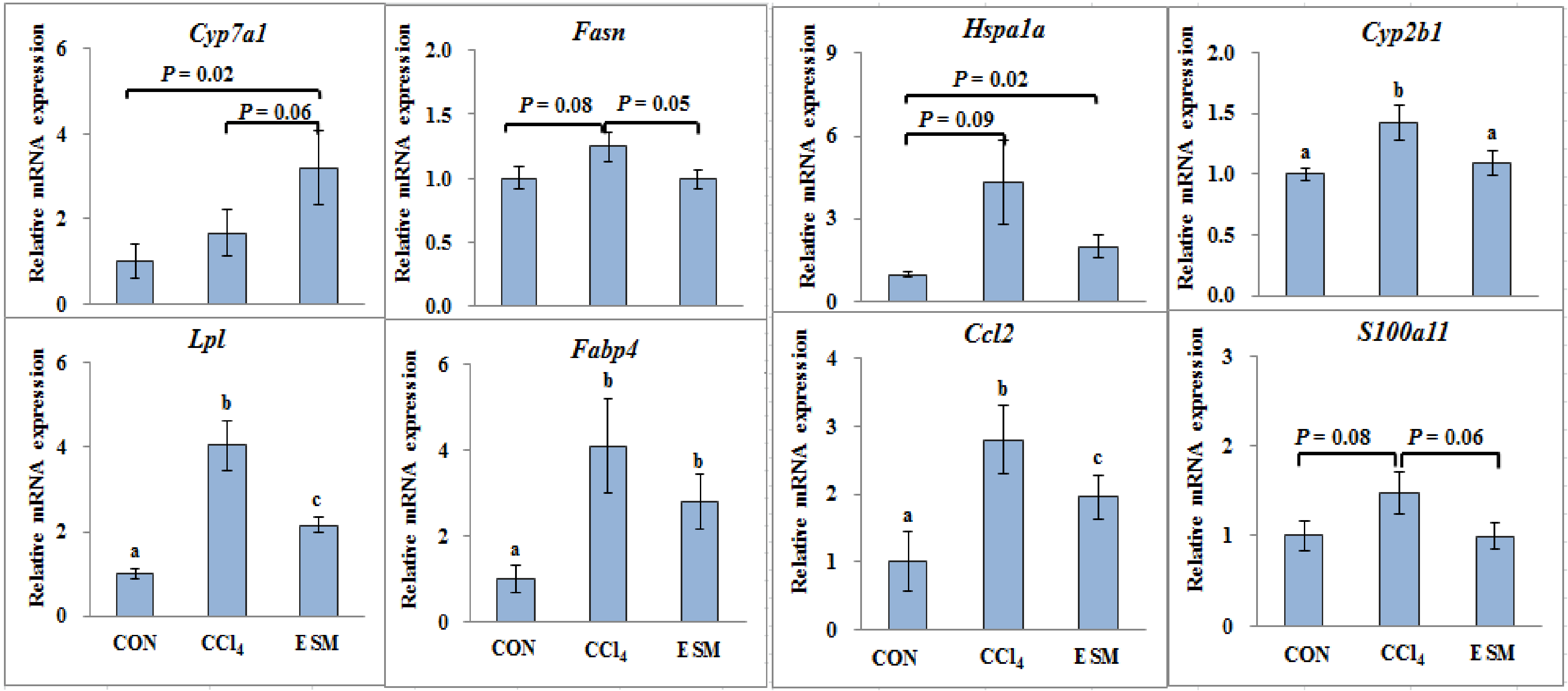

Supplementary Figure 1. Expression of genes involved in fatty acid metabolism, stress and inflammation. CON, control rats; CCl_4, rats administered CCl_4; ESM, rats administered CCl_4 and ESM (20 g kg^{-1}). Results are means ± SE in each group (n=6). Data with different letters (a,b,c) are significantly different at $P < 0.05$ by Dunnett's test.
